# Early Peri-Admission Lactate-to-Albumin (LAR), C-Reactive Protein-to-Albumin (CAR), and Procalcitonin-to-Albumin (PAR) Ratios and ICU Mortality in a Tertiary Cardiac ICU

**DOI:** 10.3390/jcm15020826

**Published:** 2026-01-20

**Authors:** Krzysztof Żerdziński, Michał Gałuszewski, Julita Janiec, Michał Skrzypek, Łukasz J. Krzych

**Affiliations:** 1 Students Department “#Intensywna_Po_Godzinach”, Department of Acute Medicine, Faculty of Medical Science in Zabrze, Medical University of Silesia, 41-800 Zabrze, Poland; kzerdzinskik@gmail.com (K.Ż.); s86715@365.sum.edu.pl (M.G.); 2 Department of Biostatistics, Faculty of Public Health in Bytom, Medical University of Silesia, 41-902 Bytom, Poland; mskrzypek@sum.edu.pl; 3 Department of Acute Medicine, Faculty of Medical Science in Zabrze, Medical University of Silesia, 41-800 Zabrze, Poland; lkrzych@sum.edu.pl; 4 Department of Anesthesiology and Intensive Care, Upper-Silesian Medical Center, 40-635 Katowice, Poland

**Keywords:** lactate, C-reactive protein, procalcitonin, albumin, intensive care unit, critical care, risk stratification, mortality, prognosis, biomarkers

## Abstract

**Background/Objectives:** Critically ill adults in intensive care units (ICUs) remain at high risk of death, while commonly used severity scores are complex and not always available at admission. We evaluated peri-admission lactate-to-albumin (LAR), C-reactive protein-to-albumin (CAR) and procalcitonin-to-albumin (PAR) ratios at ICU entry to predict ICU mortality in a cardiovascularly burdened cohort. **Methods:** We performed a single-centre retrospective observational cohort study in a tertiary cardiac ICU including adult admissions in 2024 with complete peri-admission lactate, C-reactive protein, procalcitonin and albumin. **Results:** Of 212 ICU admissions, 137 met the inclusion criteria. ICU mortality was 48.9%. Non-survivors had higher composite ratios and lower albumin than survivors. In multivariable models, LAR and CAR, but not PAR, remained independently associated with ICU mortality after adjustment for age, sex, and admission category. Receiver operating characteristic areas under the curve (AUC) were 0.692 for LAR, 0.677 for CAR and 0.625 for PAR. Cut-offs of LAR ≥ 0.106, CAR ≥ 3.18 and PAR ≥ 0.143 identified high-risk subgroups, with odds ratios for death of 6.18, 4.20 and 2.70, respectively, compared with lower-ratio patients, and LAR provided the best overall discrimination. **Conclusions:** Peri-admission LAR, CAR and PAR derived from routine tests in the ICU are associated with ICU mortality in critically ill adults, with LAR and CAR providing independent prognostic information and LAR showing the best discrimination. These simple composite ratios may complement severity scores for early risk stratification and warrant external validation.

## 1. Introduction

Critically ill adults treated in intensive care units (ICUs) remain at substantial risk of death, with reported mortality around 10–22% and reaching 25–35% in cases of sepsis. Outcomes vary between countries and units due to differences in case mix and organisation of care [1,2,3]. The early identification of high-risk patients at ICU admission is therefore central to ICU practice. However, widely used prognostic scores can be time-consuming, depend on multiple variables, and are not always immediately available, which creates interest in simple tools based on routinely collected laboratory data.

Several laboratory parameters are routinely used to reflect inflammation, infection, and organ dysfunction in acute illness. Lactate (LAC) is a marker of tissue hypoperfusion and impaired oxygen utilisation, and elevated or persistent hyperlactataemia is associated with greater shock severity and higher mortality [4,5]. C-reactive protein (CRP) is widely available and low-cost but non-specific, with prognostic utility that varies across clinical contexts [6,7,8]. Procalcitonin (PCT) is more specific for bacterial infection and can correlate with sepsis severity, yet single time-point measurements have limited value for mortality prediction and are often used to support treatment monitoring [9,10,11]. Albumin (ALB), a negative acute-phase protein, reflects inflammatory burden and nutritional status. Hypoalbuminemia is consistently associated with worse outcomes but remains non-specific [12,13,14]. In practice, these markers are most informative when interpreted together rather than in isolation [7,15,16].

To integrate information from different biological pathways, composite biomarkers that combine two or more routinely measured parameters are increasingly being studied as pragmatic prognostic tools in critical care [17,18,19]. Such indices may improve risk stratification while remaining easy to compute at the bedside, but their clinical use depends on clear definitions, population-specific evaluation, and external validation of cut-offs [19,20,21]. Among these indices, the lactate-to-albumin ratio (LAR), C-reactive protein-to-albumin ratio (CAR), and procalcitonin-to-albumin ratio (PAR) combine markers of metabolic or inflammatory stress with ALB, potentially capturing acute derangement alongside baseline physiological reserve. Higher values of these ratios have been associated with more severe disease and higher mortality in several settings [22,23,24].

For practical implementation, cut-off values must be interpreted cautiously because optimal thresholds may shift with disease prevalence, patient characteristics, and measurement timing. Consequently, cut-offs derived from a given cohort require confirmation in independent populations before they can be used as decision thresholds [25,26]. Despite these limitations, composite ratios based on routine tests remain attractive, particularly when rapid triage is needed and complex multivariable models are not readily available [19,27].

Although LAR, CAR, and PAR have each shown prognostic potential, previous studies have often focused on a single ratio or specific disease entities and rarely compare all three indices against their individual components at ICU entry, especially in cardiovascularly burdened ICU populations (e.g., post cardiac surgery or post cardiac arrest). As a result, the incremental prognostic value of these simple ratios at ICU admission in such patients remains uncertain.

Therefore, this study evaluated whether peri-admission LAR, CAR, and PAR at ICU admission are associated with ICU mortality and compared their predictive performance with that of LAC, CRP, PCT, and ALB assessed separately.

## 2. Materials and Methods

### 2.1. Study Design

A retrospective, single-centre observational cohort study was conducted, using routinely collected clinical data from index ICU admissions. The source population comprised all adult ICU admissions to the study unit between 1 January 2024 and 31 December 2024. The study was strictly non-interventional: all clinical decisions and patient management occurred as part of standard care during the 2024 ICU stays and were not influenced by the study in any way.

The study timeline consisted of two distinct phases. First, clinical care and data generation occurred during the predefined observation period (calendar year 2024). Second, data retrieval and dataset construction were performed after the completion of all eligible admissions: laboratory results for LAC, CRP, PCT, and ALB, together with sampling timestamps, were extracted from the electronic medical record and laboratory information systems between 21 March 2025 and 28 July 2025. Outcomes were obtained from the same institutional records.

To reflect the real-world availability of laboratory data around ICU admission, prognostic indices were operationalised using a prespecified early peri-admission window rather than a single “time-zero” draw. In routine care, lactate, inflammatory markers, and albumin are frequently sampled asynchronously (e.g., in the emergency department, operating room, or shortly after ICU arrival), and a complete admission-time panel is often unavailable. We therefore used a time-stamped, anchor-based approach designed to maximise data completeness while preserving temporal proximity to ICU admission.

Baseline was defined as measurements captured within the first 36 h of ICU stay. ALB served as the anchoring measurement and was required within the first 24 h. LAC/CRP/PCT were defined as the highest value within an ALB-anchored window (±12 h relative to ALB sampling), restricted to 0–36 h after ICU admission. This approach intentionally prioritises the nearest early “worst” value when markers are obtained at different times, which may better reflect early physiological derangement for risk stratification when simultaneous admission-time measurements are not available. However, because values were not necessarily obtained at a single fixed time point, comparability with studies using strictly synchronous admission-time measurements is inherently limited. All laboratory timestamps were required to be on/after the recorded ICU admission time, and any measurements obtained before ICU admission were not eligible and were excluded by design.

No repeat ICU admissions were included in the final analytical cohort, and therefore the dataset comprised 137 unique patients and intra patient correlation was not applicable.

**Figure 1:**
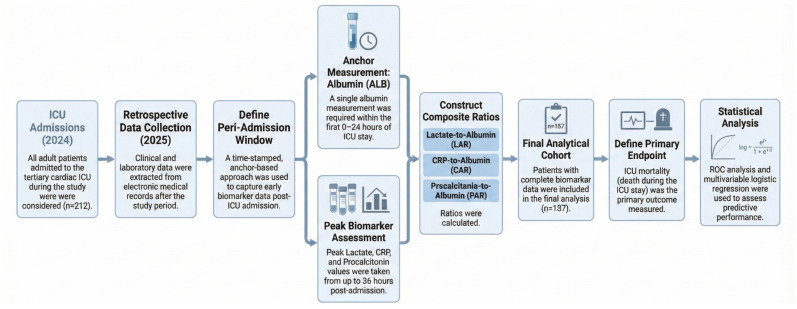
Study flow and temporal structure of the retrospective cohort.

For transparency, the study was retrospectively registered at ClinicalTrials.gov in November 2025. In February 2025, the local Bioethics Committee stated that the analysis of routinely collected clinical data does not constitute a medical experiment and does not require formal ethics committee approval, so informed consent was not applicable. This study is reported in accordance with the Strengthening the Reporting of Observational Studies in Epidemiology (STROBE) guidelines (Supplementary File S1). The study was retrospectively registered at ClinicalTrials.gov (NCT07242027). The publicly available registry record provides full methodological details. The overall study flow and the temporal structure of data collection and peri-admission measurements are summarised in Figure 1.

### 2.2. Study Setting

The study was conducted in the Cardio-Anaesthesiology ICU “B” of the Silesian Center for Heart Diseases in Zabrze, Poland. This is a tertiary referral centre providing the full spectrum of cardiology and cardiac surgery procedures, including ECMO, IABP/VAD support, and heart transplantation, with 24/7 diagnostic and therapeutic facilities. The unit operates within the structure of the Medical University of Silesia in Katowice.

### 2.3. Population, Inclusion and Exclusion Criteria

All adult patients aged 18 years or older admitted to the ICU during the study period were eligible. Included patients required availability of ALB and corresponding peri-admission biomarker window as defined. Exclusion criteria were age below 18 years, no ALB measurement within the first 24 h of ICU stay, missing LAC, CRP, or PCT within the defined window, duplicate records, or ambiguous inter-ward transfers that precluded attribution of baseline measurements to a single ICU admission.

Included admissions were assigned to six groups according to the leading diagnosis, based on discharge summaries. Each case was classified independently by at least two investigators. If classifications differed, a third investigator adjudicated, and the final assignment required agreement between two reviewers.

- (CF) Cardiovascular failure, including cardiogenic shock, STEMI, NSTEMI, post-cardiac surgery, VA ECMO.
- (CA) Post-cardiac arrest and admission due to related complications.
- (RF) Respiratory failure, including ARDS, respiratory distress, pulmonary oedema, VV ECMO, pulmonary inflammation.
- (VF) Vascular failure, including EVAR and post-vascular surgery care.
- (S) Sepsis, including urosepsis.
- (M) Mixed group, more than one leading condition at presentation.

### 2.4. Data Sources and Data Collection

Data were obtained from the AMMS electronic medical record system, linked to the laboratory information system of the Silesian Center for Heart Diseases. Demographic variables, including age and sex, dates and times of ICU admission and discharge, diagnoses from discharge summaries, and treatment outcome were extracted. Laboratory data for ALB, LAC, CRP, and PCT were extracted together with sampling dates and times to allow reconstruction of the early peri-admission measurement window.

Data extraction was performed by K.Ż., M.G., and J.J., and the team composition remained unchanged throughout the study. Patient identifiers were stored separately from the analytical dataset. A pseudonymisation key linking the hospital identification number to coded study IDs was kept in an encrypted offline file, and the working dataset contained coded IDs only and was stored in an access-restricted spreadsheet environment.

Deduplication and internal consistency checks were performed, including chronology of events and plausibility ranges, and extreme values were flagged for secondary review. Missing data were not imputed. For quality control, 10 percent of records were randomly checked against source documentation.

### 2.5. Definitions and Units of the Indices

All laboratory measurements were analysed in the units listed in Table 1. Indices were calculated using the following formulas, and all results and cut-off values are reported in these units.

$$LAR\;[\frac{mmol}{g}]=\frac{Lactate\;[mmol/L]}{Albumin\;[g/L]}$$

$$CAR\;[\frac{mg}{g}]=\frac{C-reactive\;protein\;[mg/L]}{Albumin\;[g/L]}$$

$$PAR\;[\frac{\mu g}{g}]=\frac{Procalcitonin\;[\mu g/L]}{Albumin\;[g/L]}$$

### 2.6. Endpoints

The primary endpoint was ICU mortality, defined as death during the ICU stay for each included ICU admission. Secondary endpoints included ICU length of stay, expressed in days. In addition, the predictive performance of LAR, CAR, and PAR was compared with that of LAC, CRP, PCT, and ALB assessed separately.

### 2.7. Statistical Analysis

Statistical analysis was performed using R software, version 4.5.1 (R Foundation for Statistical Computing, Vienna, Austria). Quantitative variables with a normal distribution were presented as means with standard deviation, whereas those deviating from normality were reported as medians with lower and upper quartiles. Normality was assessed using the Shapiro–Wilk test. Categorical variables were described as absolute counts and percentages.

Between-group comparisons for categorical variables were performed using the χ^2^ test. For quantitative variables, depending on distribution, Student’s *t*-test or Mann–Whitney U test was used for comparisons between two groups, and ANOVA or the Kruskal–Wallis test for comparisons across multiple groups.

The predictive ability of the analysed variables for ICU mortality was assessed using the area under the receiver operating characteristic curve (AUC, Area Under the Curve). In addition, multivariable logistic regression models were applied to estimate the risk of death, and the results were reported as odds ratios (OR) with 95% confidence intervals (CI).

Two separate multivariable logistic regression models were prespecified. Both models were adjusted for age, sex, and admission category. The first model evaluated simple biomarkers (LAC, CRP, PCT, and ALB), and the second model evaluated composite indices (LAR, CAR, and PAR). This separation was used to avoid collinearity between component biomarkers and derived ratios. Given the limited number of outcome events, adjustment was intentionally parsimonious to reduce overfitting. Therefore, residual confounding by unmeasured baseline severity, comorbidity burden, and early ICU interventions cannot be excluded. A *p*-value < 0.05 was considered statistically significant.

## 3. Results

**Figure 2:**
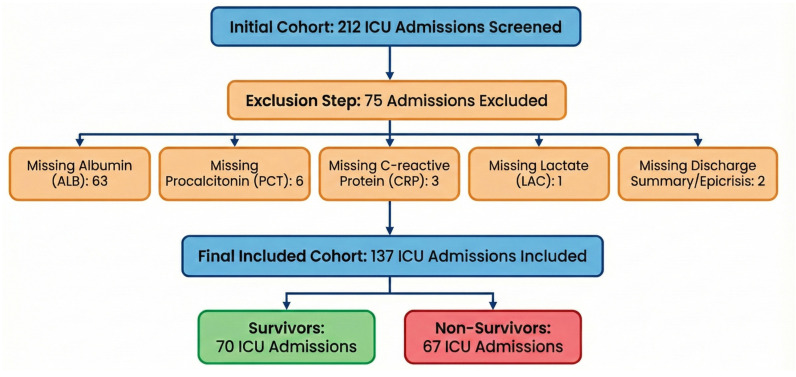
Flow diagram of ICU admission screening and inclusion, and definition of the peri-admission measurement window.

Between 1 January and 31 December 2024, 212 ICU admissions were screened. After applying predefined inclusion and exclusion criteria and excluding admissions with incomplete biomarker data required to compute the composite indices, 137 patients were included in the final analysis, as shown in Figure 2.

The excluded admissions were primarily due to missing ALB measurements (n = 63), followed by missing PCT (n = 6), CRP (n = 3), LAC (n = 1), or insufficient information in the discharge/epicrisis summary (n = 2).

### 3.1. Descriptive Statistics

Demographic and clinical characteristics of the included ICU admissions are presented in Table 2. The median age was 69 years (IQR 55–75, range 20–93), and most patients were male (70.1%). The median ICU LOS was 8 days (IQR 3–19). ICU mortality was 48.9%. The largest admissions category consisted of patients with cardiovascular failure (CF, 36.5%), followed by post-cardiac arrest patients (CA, 23.4%). The least represented groups were patients with mixed presentations (M, 5.1%) and those with sepsis or septic shock (S, 5.8%). Peri-admission measurements of LAC, CRP, PCT, and the composite indices (LAR, CAR, PAR) showed right-skewed distributions.

### 3.2. Survival-Based Comparison

The comparison of patient characteristics stratified by ICU survival is presented in Table 3. No significant differences were observed between survivors and non-survivors in terms of age, sex, or distribution of admission categories. Survivors had a longer ICU LOS than non-survivors (median 9.5 vs. 7.0 days, *p* = 0.048). Non-survivors exhibited higher median peri-admission concentrations of LAC (2.70 vs. 1.79 mmol/L, *p* = 0.001), CRP (147.6 vs. 72.4 mg/L, *p* = 0.002), and PCT (1.75 vs. 0.96 µg/L, *p* = 0.021) compared with survivors. Composite indices were also significantly elevated among non-survivors, LAR (0.11 vs. 0.05, *p* < 0.001), CAR (4.54 vs. 2.60, *p* < 0.001), and PAR (0.06 vs. 0.03, *p* = 0.012). Mean ALB concentration was lower in patients who died (29.4 vs. 32.3 g/L, *p* = 0.003).

### 3.3. Association Between Biomarkers and ICU Mortality: Logistic Regression

#### 3.3.1. Simple Biomarkers (LAC, CRP, PCT, ALB)

In multivariable logistic regression models with ICU mortality as the dependent variable, predictors included age, sex, admission category, and peri-admission simple biomarker values (Table 4). Due to the small number of cases, the mixed group (M) was excluded from the analysis. Neither age nor admission category showed a significant association with ICU mortality. Higher baseline concentrations of LAC and CRP were independently associated with an increased risk of ICU death (LAC: OR 1.19; 95% CI 1.06–1.38; *p* = 0.007; CRP: OR 1.01; 95% CI 1.00–1.01; *p* = 0.017), whereas PCT and ALB did not reach statistical significance in this model.

#### 3.3.2. Composite Biomarkers (LAR, CAR, PAR)

In an analogous model including the composite indices LAR, CAR, and PAR (Table 5), age, sex, and admission category again showed no significant association with mortality. Higher LAR and CAR values were independently associated with an increased risk of ICU death (LAR: OR 1.05; 95% CI 1.02–1.09; *p* = 0.007; CAR: OR 1.18; 95% CI 1.04–1.35; *p* = 0.014). PAR showed the weakest and least precise association with ICU mortality in this model and did not reach statistical significance (OR 1.80; 95% CI 0.79–7.60; *p* = 0.310), with wide confidence intervals indicating substantial uncertainty.

### 3.4. Discriminatory Performance—ROC Curves and Cut-Off Values

**Figure 3:**
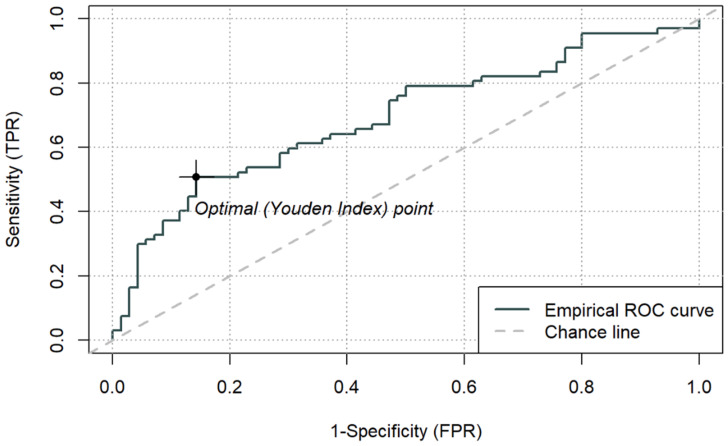
ROC curve for ICU mortality using peri-admission LAR and cut-off based on the Youden index (0.1064) (n = 137).

**Figure 4:**
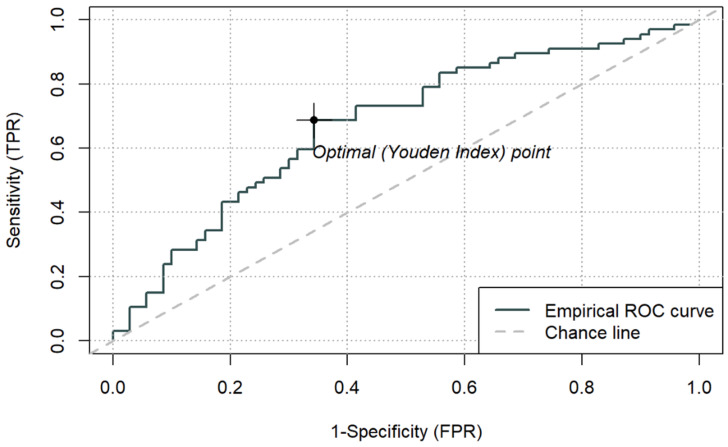
ROC curve for ICU mortality using peri-admission CAR and cut-off based on the Youden index (3.176) (n = 137).

**Figure 5:**
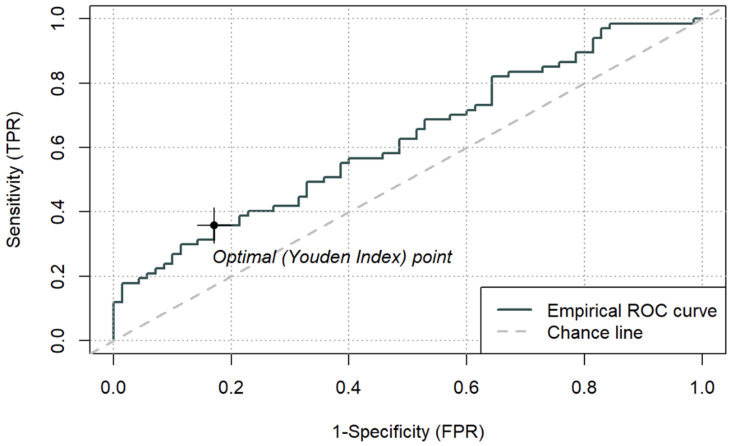
ROC curve for ICU mortality using peri-admission PAR and cut-off based on the Youden index (0.14258) (n = 137).

**Figure 6:**
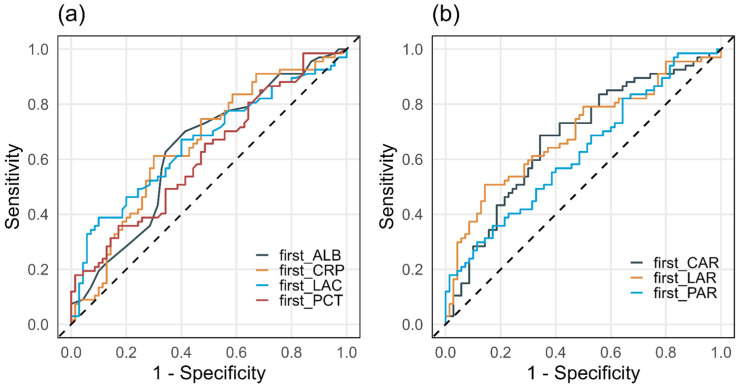
Comparison of ROC curves for ICU mortality using peri-admission simple (**a**) and composite (**b**) biomarkers (n = 137).

The discriminatory performance of simple biomarkers (LAC, CRP, PCT, ALB) and composite indices (LAR, CAR, PAR) for ICU mortality was assessed using ROC curve analysis. AUC values, optimal cut-off points based on the Youden index, and corresponding sensitivity and specificity estimates are summarised in Table 6. ROC curves for LAR, CAR, and PAR are presented in Figure 3, Figure 4 and Figure 5, while a combined comparison of simple and composite biomarkers is shown in Figure 6. Cut-off values were derived from the present dataset using the Youden index and should be interpreted as data-driven thresholds.

Among the composite indices, LAR achieved the highest AUC (0.692; 95% CI 0.603–0.781), with an optimal cut-off of 0.1064. For LAR ≥ 0.1064, ICU mortality reached 77.3% (*p* < 0.001). CAR demonstrated an AUC of 0.677 (95% CI 0.586–0.767), with an optimal cut-off of 3.176, above which mortality was 65.7% (*p* < 0.001). PAR yielded a lower AUC (0.625, 95% CI 0.531–0.718), with an optimal cut-off of 0.143, which identified a subgroup with higher observed 66.7% mortality (*p* = 0.013), while PAR was not an independent predictor in the continuous multivariable model. Sensitivity and specificity for the optimal thresholds of LAR, CAR, and PAR ranged from 0.36 to 0.69 and 0.66–0.86, respectively.

### 3.5. Odds Ratios for Mortality Based on ROC-Derived Cut-Off Values

Odds ratios for ICU mortality above the optimal cut-off points for LAR, CAR, and PAR, derived from ROC analysis, are presented in Table 7. Exceeding the LAR threshold was associated with approximately six-fold higher odds of death (OR 6.18; 95% CI 2.80–14.68; *p* < 0.001). For CAR, the odds of death increased roughly four-fold (OR 4.20; 95% CI 2.08–8.72; *p* < 0.001). PAR showed a significant but weaker association, with a 2.7-fold increase in the odds of ICU death above its cut-off (OR 2.70; 95% CI 1.24–6.15; *p* = 0.015).

### 3.6. Comparison Across ICU Admission Categories

A comparison of age, ICU LOS, mortality, and peri-admission biomarker values across admission groups is presented in Table 8. Age differed significantly between groups (*p* < 0.001), with lower median age in the RF and S categories compared with other groups. ICU LOS also varied significantly (*p* < 0.001), with the longest median stay in the RF group (16 days, IQR 7–28) and CF group (12 days, IQR 4–20), and the shortest in the VF group (3 days, IQR 1–6). Mortality rates and sex distribution did not differ significantly between admission groups (*p* = 0.10 and *p* > 0.9, respectively).

Median peri-admission concentrations of LAC, PCT, ALB, and composite indices (LAR, CAR, PAR) differed significantly between groups (all *p* < 0.05), whereas CRP did not (*p* = 0.12). The highest PCT concentrations and composite index values were observed in the sepsis group, while mixed and septic patients demonstrated the lowest ALB levels. Due to the small sample sizes in the M and S groups, results for these categories should be interpreted with caution.

### 3.7. Sex-Based Comparison

A comparison of clinical variables and peri-admission biomarker values between women and men is summarised in Table 9. No significant differences were observed in age or ICU LOS. Most simple and composite biomarkers showed comparable distributions across sexes, with the exception of CRP, which was higher in men (median 112.96 vs. 82.71 mg/L, *p* = 0.047).

### 3.8. Correlations Between Composite Indices and Other Variables

Correlations between age, sex, and composite indices (LAR, CAR, PAR) are presented in Table 10. Most correlation coefficients were weak and close to zero. Moderate positive correlations were observed between LAR and PAR (cor = 0.33, *p* < 0.001) and between CAR and PAR (cor = 0.43, *p* < 0.001), indicating partial but incomplete overlap in the information captured by these indices. No clear associations were observed between composite indices and age or sex. Sex was treated as a binary variable for correlation analysis.

## 4. Discussion

This study assessed three routinely available composite ratios (LAR, CAR, PAR) in a tertiary cardiac ICU cohort and benchmarked their prognostic performance against the corresponding single biomarkers. Using prespecified early peri-admission measurements, LAR and CAR showed the most consistent associations with ICU mortality, whereas PAR demonstrated a weaker and less stable signal.

### 4.1. Comparison with Previous Studies on LAR, CAR and PAR in Critically Ill Patients

Interpretation of sepsis-specific findings is limited in this cohort because the Sepsis and Mixed categories were small. Therefore, comparisons to septic-dominant validation studies should be treated cautiously.

#### 4.1.1. LAR, CAR and PAR in General Critically Ill Populations

In heterogeneous ICU populations, LAR and CAR have repeatedly shown reproducible associations with mortality. In MIMIC-III (n = 6414), LAR achieved an AUC of 0.69 and outperformed lactate alone for ICU mortality prediction [28]. In a nationwide Japanese cohort (n = 234,774), LAR yielded an AUC of 0.761 for in-hospital mortality [29]. CAR has similarly shown consistent adverse-outcome associations, including dose–response patterns and ROC-derived cut-offs across cohorts [30,31,32,33]. In a large risk-modelling study (n = 19,720), LAR and CAR ranked among the strongest biomarkers after SOFA, and parsimonious models incorporating LAR-based indices achieved AUCs comparable to SOFA and APACHE II [34].

Within this cardiac ICU cohort, discrimination was moderate (AUC 0.692 for LAR, 0.677 for CAR, 0.625 for PAR), and ROC-derived thresholds identified subgroups with substantially higher ICU mortality. In adjusted models, LAR and CAR remained independently associated with ICU mortality, whereas PAR did not retain significance as a continuous predictor, consistent with a weaker and less robust prognostic contribution of PCT-based ratios in this setting.

#### 4.1.2. LAR, CAR and PAR in Cardiology and Cardiac Intensive Care Populations

In cardiology cohorts, elevated LAR has been associated with higher short- and longer-term mortality. In acute myocardial infarction, LAR has discriminated survivors from non-survivors with AUCs around 0.73 and independently predicted mortality across multiple time horizons [35,36]. Graded risk increases across LAR strata have been reported in coronary artery disease and heart failure [37,38,39]. In hypertensive ICU patients, the highest LAR tertile was linked to higher 28-day mortality with a non-linear relationship and partial mediation via severity scores [40]. After cardiac arrest, higher LAR has been associated with worse survival and neurological outcomes [41,42].

This cohort, dominated by circulatory failure, post-cardiac arrest, and post-cardiac surgery admissions, is clinically aligned with these populations. The observed AUCs were lower than in some cardiology-only analyses, plausibly reflecting greater diagnostic heterogeneity and high baseline risk, as well as differences in sampling definitions relative to single time-point admission draws or longitudinal maxima/means.

#### 4.1.3. LAR, CAR and PAR in Respiratory Failure and ARDS Populations

In respiratory failure and ARDS cohorts, LAR and CAR have also demonstrated prognostic value, and PAR has been reported as informative in more homogeneous respiratory ICU settings and in multi-marker models [43,44,45,46]. In this predominantly cardiological cohort, CAR and PAR separated risk groups in cut-off-based analyses. However, the lower AUC for PAR and its lack of significance in the continuous adjusted model suggest that PAR may be more context-dependent and less transportable across ICU case mixes.

#### 4.1.4. Synthesis Across Populations and Implications for Our ICU Cohort

Across prior evidence, LAR and CAR appear consistently mortality-associated, whereas PAR shows more variable performance. In this tertiary cardiac ICU cohort, LAR and CAR provided the most reliable early risk classification signal, while PAR should be interpreted cautiously. Because cut-offs were data-driven and models lacked external validation and comprehensive adjustment for severity and early interventions, these findings should be treated as hypothesis-supporting and require confirmation in independent cardiac ICU cohorts.

### 4.2. Strengths of the Study, Limitations and Future Directions

#### 4.2.1. Strengths of the Study

Strengths include a consecutive one-year cohort from a tertiary cardio-anaesthesiology ICU, an objective endpoint (ICU mortality), and laboratory measurements processed within a single institutional laboratory with documented quality control. Composite indices were explicitly defined, analysed alongside individual components, and evaluated using prespecified multivariable logistic regression. Discrimination was quantified by ROC analysis with transparent cut-off derivation.

#### 4.2.2. Limitations

Key limitations include the retrospective single-centre design and the resulting susceptibility to residual confounding and documentation bias. Generalisability is constrained by the specialised cardiac ICU setting and the modest sample size, with small diagnostic subgroups limiting subgroup precision. Accordingly, subgroup analyses for conditions that were infrequent in this setting (particularly sepsis) were not pursued, as they would be underpowered and potentially misleading; importantly, our focus on a cardiologically burdened ICU population was intentional and constitutes a key aspect of the study’s novelty.

Selection bias is possible because many admissions were excluded due to missing biomarker measurements required to compute the ratios. In routine ICU workflows, missingness is unlikely to be random, because ordering patterns and sampling intensity may vary by presentation, perceived severity, timing, pre-ICU workup location, early death or transfer, and logistics. As a result, included admissions may over-represent patients with more complete early laboratory monitoring, which may affect baseline risk and apparent performance and limit the transportability of derived cut-offs. A formal included-versus-excluded comparison was not feasible. Therefore, applicability is most defensible for patients in whom the required biomarkers are obtained early.

Biomarkers were derived using an early peri-admission, anchor-based window and peak values rather than strictly synchronous admission-time sampling, which limits direct comparability with studies using fixed-time admission draws and may shift effect estimates by accentuating early severity signals. In addition, the timing and frequency of serial measurements were not standardised across admissions, which precluded robust assessment of lactate kinetics (e.g., delta/clearance within 24–36 h) and may have contributed to heterogeneity in “peak” values captured within the anchor window.

Severity scores and detailed comorbidity and treatment variables were not incorporated, so even adjusted associations should not be interpreted causally. In particular, validated ICU severity scores (e.g., SOFA or APACHE II) were not routinely recorded in a uniform, extractable format across the cohort (with heterogeneous documentation across electronic and paper records), and reliable retrospective reconstruction was not feasible without introducing additional missingness and misclassification. Similarly, detailed haemodynamic treatment data, including vasopressor exposure, dosing, and escalation within the first 24 h, were not consistently available and could not be incorporated without further reducing the analysable sample and increasing selection bias. To avoid collinearity, raw biomarkers and ratios were not combined in a single multivariable model, which precludes direct “incremental value” testing of ratios over components in a unified specification. Moreover, we did not have access to systematically recorded anthropometric or nutritional status variables (e.g., BMI, malnutrition/cachexia indicators), which may confound albumin-based associations and warrants dedicated investigation in future, prospectively phenotyped cohorts. Finally, discrimination was moderate, and models were not internally or externally validated. Youden-derived cut-offs should be considered cohort-specific.

#### 4.2.3. Future Directions

Future work should validate these findings in prospective, multicentre cohorts with broader ICU case mixes and should report both discrimination and calibration. Studies should assess incremental value over established severity scores and incorporate key comorbidities and early interventions, ideally within a validation framework and with clinical utility evaluation. Longitudinal trajectories during ICU stay may clarify whether dynamic changes add prognostic information beyond early measurements. If robustness and added value are confirmed, implementation-focused studies should evaluate whether integration into decision support improves care processes and outcomes.

## 5. Conclusions

In this tertiary cardiac ICU cohort, early peri-admission LAR, CAR, and PAR derived from routine laboratory tests showed prognostic separation for ICU mortality. In multivariable models adjusted for age, sex, and admission category, LAR and CAR remained independently associated with ICU death and showed the strongest overall signal, whereas PAR did not reach statistical significance as a continuous predictor. Discrimination was moderate, and ROC-derived cut-offs were data-driven and should be interpreted as cohort-specific. These simple indices may complement, but not replace, established severity assessment, and require confirmation in larger, multi-centre cohorts with validation and richer clinical adjustment.

## Figures and Tables

**Table 1:** Reference laboratory ranges and units of collected data.

	Reference Range	Unit
LAC	0.5–2.0	mmol/L
CRP	<5	mg/L
PCT	<0.5	μg/L
ALB	32–48	g/L

**Table 2:** Demographic and clinical characteristics of included ICU admissions (n = 137).

Treatment Outcome					
Variable	Min	Q1	Median	Q3	Max
Age, years	20	55	69	75	93
LOS in ICU, days	1	3	8	19	97
Sex					
Female	41		29.9%		
Male	96		70.1%		
ICU mortality					
Survivor	70		51.1%		
Non-Survivor	67		48.9%		
Group					
CF	50		36.5%		
CA	32		23.4%		
RF	21		15.3%		
VF	19		13.9%		
S	8		5.8%		
M	7		5.1%		
**Peri-admission biomarkers**					
Variable	Min	Q1	Median	Q3	Max
LAC	0.55	1.51	2.08	3.50	21.74
CRP	0.50	45.01	102.70	184.41	441.64
PCT	0.03	0.30	1.29	4.51	311.44
ALB	18.00	27.00	31.00	35.00	45.00
LAR	0.0145	0.0462	0.067	0.131	0.75
CAR	0.0139	1.1845	3.237	6.414	16.65
PAR	0.0007	0.0107	0.047	0.160	11.53

n (%); Median (Q1, Q3).

**Table 3:** Survival-based comparison of clinical and peri-admission laboratory parameters.

Characteristic	Survivor (n = 70)	Non-Survivor (n = 67)	*p*-Value *
Age, years	69.00 (51.00, 76.00)	68.00 (58.00, 74.00)	0.8
LOS in ICU, days	9.50 (5.00, 22.00)	7.00 (3.00, 16.00)	0.048
Sex			>0.9
Female	21 (30.0%)	20 (29.9%)	
Male	49 (70.0%)	47 (70.1%)	
Group			0.10
CF	25 (35.7%)	25 (37.3%)	
CA	15 (21.4%)	17 (25.4%)	
RF	16 (22.9%)	5 (7.5%)	
VF	10 (14.3%)	9 (13.4%)	
S	2 (2.9%)	6 (9.0%)	
M	2 (2.9%)	5 (7.5%)	
**Peri-admission biomarkers**			
LAC	1.79 (1.31, 2.73)	2.70 (1.72, 6.71)	0.001
CRP	72.43 (26.78, 153.94)	147.57 (65.12, 215.65)	0.002
PCT	0.96 (0.24, 3.30)	1.75 (0.42, 7.83)	0.021
ALB, mean (SD)	32.30 (5.84)	29.39 (5.50)	0.003
LAR	0.05 (0.04, 0.08)	0.11 (0.06, 0.25)	<0.001
CAR	2.60 (0.85, 4.86)	4.54 (2.30, 7.51)	<0.001
PAR	0.03 (0.01, 0.10)	0.06 (0.01, 0.28)	0.012

n (%); Median (Q1, Q3); mean (SD). * Pearson’s Chi-squared test; Fisher’s exact test; Wilcoxon rank sum test; Welch two-sample *t*-test.

**Table 4:** Logistic regression for ICU mortality using peri-admission LAC, CRP, PCT, and ALB (included ICU admissions, n = 137).

Characteristic	OR	95% CI	*p*-Value
Age	1.017	0.989, 1.048	0.253
Group, n (%)			
CF (n = 50)	0.680	0.083, 4.130	0.686
CA (n = 32)	0.856	0.096, 5.860	0.878
RF (n = 21)	0.357	0.035, 2.722	0.339
VF (n = 19)	0.419	0.046, 2.912	0.395
S (n = 8)	1.397	0.096, 20.577	0.801
Sex			
Female	—	—	
Male	0.747	0.314, 1.763	0.505
**Peri-admission biomarkers**			
LAC	1.192	1.063, 1.375	0.007
CRP	1.006	1.001, 1.010	0.017
PCT	1.024	0.992, 1.085	0.318
ALB	0.954	0.884, 1.027	0.217

Abbreviations: CI = Confidence interval, OR = odds ratio.

**Table 5:** Logistic regression for ICU mortality using peri-admission LAR, CAR, and PAR (included ICU admissions, n = 137).

Characteristic	OR	95% CI	*p*-Value
Age	1.018	0.990, 1.048	0.231
Group			
CF (n = 50)	0.666	0.082, 4.083	0.669
CA (n = 32)	0.803	0.093, 5.392	0.826
RF (n = 21)	0.342	0.034, 2.602	0.316
VF (n = 19)	0.415	0.045, 2.941	0.393
S (n = 8)	1.356	0.092, 20.397	0.819
Sex			
Female	—	—	
Male	0.748	0.319, 1.740	0.500
**Peri-admission biomarkers**			
LAR	1.048	1.017, 1.090	0.007
CAR	1.179	1.038, 1.352	0.014
PAR	1.800	0.792, 7.598	0.310

Abbreviations: CI = Confidence interval, OR = odds ratio.

**Table 6:** ROC analysis for ICU mortality using peri-admission composite biomarkers (LAR, CAR, PAR) (included ICU admissions, n = 137).

Biomarker	AUC (95% CI)	Cut-Off	Accuracy (95% CI)	Sensitivity (95% CI)	Specificity (95% CI)
LAR	0.692 (0.603–0.781)	0.1064	0.686 (0.62–0.759)	0.507 (0.382–0.632)	0.857 (0.753–0.929)
CAR	0.677 (0.586–0.767)	3.176	0.672 (0.606–0.752)	0.687 (0.562–0.794)	0.657 (0.534–0.767)
PAR	0.625 (0.531–0.718)	0.14258	0.599 (0.569–0.686)	0.358 (0.245–0.485)	0.829 (0.72–0.908)

**Table 7:** Odds ratios for ICU mortality above ROC-derived cut-off points (included ICU admissions, n = 137).

Characteristic	OR	95% CI	*p*-Value
LAR	6.182	2.797, 14.680	<0.001
CAR	4.198	2.082, 8.721	<0.001
PAR	2.698	1.235, 6.150	0.015

Abbreviations: CI = Confidence interval, OR = odds ratio.

**Table 8:** Clinical characteristics and peri-admission biomarker values across ICU admission groups (n = 137 ICU admissions).

Characteristic	M (n = 7)	CF (n = 50)	VF (n = 19)	RF (n = 21)	CA (n = 32)	S (n = 8)	*p*-Value *
Age, years	72 (62, 76)	69.5 (58, 76)	73 (68, 76)	52 (41, 64)	68 (60, 76)	54.5 (41, 63)	<0.001
Non-Survivor	5 (71.4%)	25 (50.0%)	9 (47.4%)	5 (23.8%)	17 (53.1%)	6 (75.0%)	0.10
LOS, days	9 (3, 21)	12 (4, 20)	3 (1, 6)	16 (7, 28)	8 (3, 11)	6.5 (3.5, 47)	<0.001
Sex, n (%)							>0.9
Female	2 (28.6%)	15 (30.0%)	6 (31.6%)	8 (38.1%)	8 (25.0%)	2 (25.0%)	
Male	5 (71.4%)	35 (70.0%)	13 (68.4%)	13 (61.9%)	24 (75.0%)	6 (75.0%)	
**Peri-admission biomarkers**							
LAC	2.98 (1.16, 7.30)	2.21 (1.70, 3.51)	2.07 (1.58, 7.34)	1.57 (1.23, 1.64)	2.71 (1.78, 5.25)	3.42 (1.37, 6.16)	0.005
CRP	159.17 (84.89, 227.64)	92.50 (49.53, 197.63)	116.89 (29.31, 169.99)	108.96 (50.34, 181.70)	65.84 (21.79, 123.85)	196.61 (62.70, 290.16)	0.12
PCT	2.09 (0.20, 4.42)	1.26 (0.39, 3.83)	0.45 (0.07, 4.11)	1.04 (0.19, 2.62)	1.49 (0.33, 6.96)	12.45 (5.55, 35.40)	0.007
ALB	27 (22, 30)	31 (28, 34)	27 (24, 34)	34 (29, 36)	33 (28, 36.5)	26.5 (24, 31)	0.004
LAR	0.11 (0.04, 0.24)	0.08 (0.05, 0.13)	0.07 (0.05, 0.33)	0.05 (0.04, 0.06)	0.08 (0.05, 0.18)	0.12 (0.05, 0.29)	0.003
CAR	5.29 (2.93, 7.59)	3.11 (1.72, 6.34)	4.16 (1.33, 6.34)	4.04 (1.18, 6.73)	2.22 (0.70, 5.02)	7.34 (2.47, 12.31)	0.043
PAR	0.07 (0.01, 0.14)	0.04 (0.01, 0.11)	0.01 (0.00, 0.11)	0.04 (0.01, 0.06)	0.05 (0.01, 0.20)	0.51 (0.18, 1.59)	0.009

n (%); Median (Q1, Q3); mean (SD). * Fisher’s exact test; Kruskal–Wallis rank sum test; One-way analysis of means (not assuming equal variances).

**Table 9:** Sex-based comparison of clinical characteristics and peri-admission biomarker values (n = 137 ICU admissions).

Characteristic	Female	Male	*p*-Value *
Age	72.00 (47.00, 77.00)	67.50 (57.00, 73.50)	0.3
LOS	8.00 (4.00, 15.00)	8.50 (3.00, 20.00)	0.8
**Peri-admission biomarkers**			
LAC	2.33 (1.57, 3.21)	1.92 (1.49, 3.97)	0.4
CRP	82.71 (28.84, 150.76)	112.96 (53.86, 208.45)	0.047
PCT	0.73 (0.27, 3.30)	1.53 (0.35, 5.44)	0.2
ALB, mean (SD)	30.29 (5.41)	31.13 (6.02)	0.4
LAR	0.08 (0.05, 0.11)	0.06 (0.05, 0.13)	0.4
CAR	3.72 (1.75, 6.54)	4.62 (2.39, 6.95)	0.3
PAR	0.03 (0.01, 0.10)	0.05 (0.01, 0.19)	0.2

n (%); Median (Q1, Q3); mean (SD). * Fisher’s exact test; Wilcoxon rank sum test; Pearson’s Chi-squared test; Welch’s two-sample *t*-test.

**Table 10:** Correlation matrix between age, sex, and composite indices among included ICU admissions (n = 137).

	Age	Sex	LAR	CAR	PAR
Age	1.000	−0.089	0.0930	−0.0380	−0.022
Sex	−0.089	1.000	−0.0740	0.1600	0.099
LAR	0.093	−0.074	1.000	0.0042	0.330
CAR	−0.038	0.160	0.0042	1.000	0.430
PAR	−0.022	0.099	0.3300	0.430	1.000

## Data Availability

The data presented in this study are available from the scientific supervisor (Ł.J.K.) of the project upon reasonable request.

## Supplementary Materials

The following supporting information can be downloaded at https://www.mdpi.com/article/10.3390/jcm15020826/s1, Supplementary File S1: STROBE Checklist.

## Abbreviations

LAR	Lactate-to-albumin ratio
CAR	C-Reactive Protein-to-albumin ratio
PAR	Procalcitonin-to-albumin ratio
ICU	Intensive Care Unit
LAC	Lactate
CRP	C-Reactive Protein
PCT	Procalcitonin
ALB	Albumin
CF	Cardiovascular failure (group)
CA	Post-cardiac arrest and hospitalised due to its complications (group)
RF	Respiratory failure (group)
VF	Vascular failure (group)
S	Sepsis (group)
M	Mixed (group)
LOS	Length of stay
AUC	Area Under the Curve
OR	Odds ratios
CI	Confidence intervals
