# Supplementary material for: Early Peri-Admission Lactate-to-Albumin (LAR), C-Reactive Protein-to-Albumin (CAR), and Procalcitonin-to-Albumin (PAR) Ratios and ICU Mortality in a Tertiary Cardiac ICU
Source: J Clin Med. 2026 Jan 20;15(2):826. doi: 10.3390/jcm15020826 (PMC12841677; doi:10.3390/jcm15020826)
Supplement: Supplementary file 1 [file jcm-15-00826-s001.zip › jcm-4039261-supplementary.pdf]

STROBE Statement—Checklist of items that should be included in reports of ***cohort studies***

	Item No	Recommendation	Informations
**Title and abstract**	1	(*a*) Indicate the study's design with a commonly used term in the title or the abstract	Title; Abstract (Methods): 'retrospective observational cohort study'.
		(*b*) Provide in the abstract an informative and balanced summary of what was done and what was found	Abstract (Background/Methods/Results/Conclusions).
**Introduction**			
Background/rationale	2	Explain the scientific background and rationale for the investigation being reported	Introduction (paras 1–4).
Objectives	3	State specific objectives, including any prespecified hypotheses	End of Introduction (final paragraph): study aim and comparisons.
**Methods**			
Study design	4	Present key elements of study design early in the paper	Methods 2.1 (Study design).
Setting	5	Describe the setting, locations, and relevant dates, including periods of recruitment, exposure, follow-up, and data collection	Methods 2.1 (dates 1 Jan–31 Dec 2024; extraction 21 Mar–28 Jul 2025) and 2.2 (ICU setting).
Participants	6	(*a*) Give the eligibility criteria, and the sources and methods of selection of participants. Describe methods of follow-up	Methods 2.3 (eligibility, exclusions, selection) and 2.6 (follow-up to ICU discharge/death). Figure 2.
		(*b*) For matched studies, give matching criteria and number of exposed and unexposed	Not applicable (no matching).
Variables	7	Clearly define all outcomes, exposures, predictors, potential confounders, and effect modifiers. Give diagnostic criteria, if applicable	Methods 2.5 (LAR/CAR/PAR definitions), 2.6 (outcome), 2.7 (covariates/confounders: age, sex, admission category).
Data sources/ measurement	8*	For each variable of interest, give sources of data and details of methods of assessment (measurement). Describe comparability of assessment methods if there is more than one group	Methods 2.4 (data sources, extraction, QC) and 2.5 (laboratory units).
Bias	9	Describe any efforts to address potential sources of bias	Methods 2.4 (deduplication/QC), 2.7 (parsimonious adjustment; residual confounding acknowledged); Discussion 4.2.2 (selection bias, confounding).
Study size	10	Explain how the study size was arrived at	Methods 2.1 (consecutive one-year cohort) and Results 3 (screened 212, included 137). No a priori sample size calculation.
Quantitative variables	11	Explain how quantitative variables were handled in the analyses. If applicable, describe which groupings were chosen and why	Methods 2.7 (continuous handling; distribution summaries; ROC Youden cut-offs) and Results 3.4–3.5 (cut-offs).
Statistical methods	12	(*a*) Describe all statistical methods, including those used to control for confounding	Methods 2.7 (tests, logistic regression, ROC; covariates adjusted).
		(*b*) Describe any methods used to examine subgroups and interactions	Results 3.6–3.8 (group, sex, correlations). No interaction terms reported.
		(*c*) Explain how missing data were addressed	Methods 2.4 (complete-case inclusion; no imputation) and Results 3 (exclusions due to missing biomarkers).
		(*d*) If applicable, explain how loss to follow-up was addressed	Not applicable (no loss to follow-up for in-ICU outcome).
		(*e*) Describe any sensitivity analyses	Not reported (no sensitivity analyses).

Results

Participants	13*	(a) Report numbers of individuals at each stage of study—eg numbers potentially eligible, examined for eligibility, confirmed eligible, included in the study, completing follow-up, and analysed	Results 3 (counts) and Figure 2.
		(b) Give reasons for non-participation at each stage	Results 3 (reasons for exclusion) and Figure 2.
		(c) Consider use of a flow diagram	Figure 2.
Descriptive data	14*	(a) Give characteristics of study participants (eg demographic, clinical, social) and information on exposures and potential confounders	Results Table 2 (baseline characteristics) and Tables 8–10 (other descriptive comparisons).
		(b) Indicate number of participants with missing data for each variable of interest	Methods 2.4 and Results 3 (excluded due to missing biomarkers). No missing data in analytic cohort after complete-case inclusion.
		(c) Summarise follow-up time (eg, average and total amount)	Results Table 2 (ICU LOS) and Methods 2.6 (follow-up definition).
Outcome data	15*	Report numbers of outcome events or summary measures over time	Results Table 2 (ICU mortality n=67) and Table 3.
Main results	16	(*a*) Give unadjusted estimates and, if applicable, confounder-adjusted estimates and their precision (eg, 95% confidence interval). Make clear which confounders were adjusted for and why they were included	Results Table 3 (unadjusted comparisons) and Tables 4–5 (adjusted OR with 95% CI); Methods 2.7 (why adjusted).
		(*b*) Report category boundaries when continuous variables were categorized	Results Table 6 (Youden cut-offs) and Table 7 (dichotomised OR).
		(*c*) If relevant, consider translating estimates of relative risk into absolute risk for a meaningful time period	Results 3.4–3.5 (mortality percentages above cut-offs).
Other analyses	17	Report other analyses done—eg analyses of subgroups and interactions, and sensitivity analyses	Results 3.6–3.8; Figures 2–5; Tables 6–10.
**Discussion**			
Key results	18	Summarise key results with reference to study objectives	Discussion 4 (opening paragraphs) and Conclusions 5.
Limitations	19	Discuss limitations of the study, taking into account sources of potential bias or imprecision. Discuss both direction and magnitude of any potential bias	Discussion 4.2.2.
Interpretation	20	Give a cautious overall interpretation of results considering objectives, limitations, multiplicity of analyses, results from similar studies, and other relevant evidence	Discussion 4.1–4.2; Conclusions 5.
Generalisability	21	Discuss the generalisability (external validity) of the study results	Discussion 4.2.2–4.2.3.
**Other information**			
Funding	22	Give the source of funding and the role of the funders for the present study and, if applicable, for the original study on which the present article is based	Backmatter

*Give information separately for exposed and unexposed groups.

Note: An Explanation and Elaboration article discusses each checklist item and gives methodological background and published examples of transparent reporting. The STROBE checklist is best used in conjunction with this article (freely available on the Web sites of PLoS Medicine at http://www.plosmedicine.org/, Annals of Internal Medicine at http://www.annals.org/, and Epidemiology at http://www.epidem.com/). Information on the STROBE Initiative is available at http://www.strobe-statement.org.
